# Abstract of the Proceedings of the Buffalo Medical Association

**Published:** 1865-05

**Authors:** Joseph A. Peters

**Affiliations:** Sec’y.


ART. II—*Abstract of the Proceedings of the Buffalo Medical Association.*

Tuesday Evening, April 4, 1865.

The Society met pursuant to adjournment, the President in the chair. Present, Drs. Lockwood, Rochester, Eastman, Gay, Gould, Congar, Wyckoff, Wetmore, Ring, Cronyn, and Trowbridge.

The Secretary being absent Dr. Ring was elected Secretary *pro tempore.* The first business in order was the election of officers, which passed off without serious breach of the peace, and with the following result:

| | |
|---|---|
| Dr. Wm. Ring, . . . . | President. |
| " Wm. Gould, . . . | Vice President. |
| " J. A. Peters, . . . | Secretary. |
| " T. T. Lockwood, . . | Treasurer. |
| " J. B. Samo, . . . . | Librarian. |

Dr. Ring having taken the chair, Dr. Samo was duly installed as Secretary *pro tempore.*

*Dr. Rochester* said that the attention of the profession, in various sections, had been called to the consideration of the disease called "Spotted Fever," and its relation to cerebro-spinal meningitis. He could not say that the diseases were identical, but they certainly appeared to possess many points in common. He had seen but two cases in this city, and they were not attended with the maculæ. The first occurred in Jackson street, in the person of a lad named Neil, about ten years of age. He was called to him on the eighth of January, in the evening. The boy had been ill for a few hours, and was screaming with headache. The attack was regarded as necralgic, the result of exposure to cold. A hot foot-bath and Dover's powder was prescribed. The next morning,

(9th,) there was no improvement. The same pain in the head continued; the face was flushed, the head was slightly drawn back, pain in the limbs was also observed as well as bronchial râles. The pupils were dilated, and did not contract on exposure to light. The diagnosis of the preceding evening was evidently incorrect, and the grave nature of the attack was apparent. A cathartic dose of calomel was ordered. Sinapisms were applied along the spinal column, and quinine was directed in doses of two grains, every two hours. In the evening the cathartic operated freely. Quinine, with the addition of a teaspoonful of whisky with each dose was continued, and Dover's powder directed in small doses at intervals of from four to six hours. On the 10th there was manifest improvement. Treatment continued. On the 11th Dr. Rochester's services were summarily dispensed with.—*(More Hibernico.)* He learned, however, subsequently, from the gentleman who succeeded him, (Dr. Cronyn,) that the lad was quite ill for several days; that the disease was evidently cerebro-spinal; that essentially the same treatment was continued, and that pretty prompt recovery took place. About this time, however, a younger child took ill, had a purpuric eruption, and was moribund when the Doctor was first called to see him.

The second case, Dr. Rochester continued, was one of sad and painful interest from many causes. On the afternoon of the first of April he was called to see a little boy five years old, the nephew of a much esteemed friend and colleague, (not resident in the city.) The history in brief was as follows: The child was healthy and full of life and animation. The preceding evening he complained slightly of fatigue, slept well however, and rose the next morning. Yet as he appeared somewhat indisposed his mother gave him a slight relaxative, (castor oil.) At 1 P. M. he seemed much more ill, and Dr. Rochester was sent for. Professional engagements prevented him from reaching him until nearly 5 P. M. At that hour he found him in an unconscious state, or rather in a condition where he had lost a portion of his faculties. It was very apparent that he could neither see nor hear; usually a very docile and amiable child, he could not be persuaded to open his mouth. He kept the teeth constantly clenched, and screamed through them when stirred or moved. His face was flushed; the eyes were open and staring; the pupils were dilated and entirely insensible to light. The hands and feet were cold; the pulse was about 96, but irregular; the respiralion was 48 per minute; the head was very slightly drawn backward. A careful exploration of the thorax and abdominal cavities revealed no cause for the serious disturbance of the system, and the condition of the organs of sight and hearing, pointed, but too distinctly to the great nervous centres. Sinapisms were applied freely to the whole surface of the body.—Cathartics and anti-spasmodic injections were administered, and eight grains of calomel were given by the mouth. Quinine in doses of two grains was ordered every three hours, and whisky one teaspoonful every hour. At 10 P. M. there was no improvement, the pulse had run up to 140. the respiration was 50. Reaction had taken place and the head and feet had become warm. Treatment continued with directions to repeat the calomel should no catharsis occur by midnight.

April 2d, 8 A. M. The bowels acted freely in the night, but no relief followed. The muscles of the upper and lower extremities are in *tonic* spasm. The pulse cannot be counted. The surface is pale and clammy. 9 A. M. died.

Dr. Rochester stated that he hoped most sincerely that this was an imported and not an indigenous case of this terrible disorder; the little boy had been to Rochester and to Medina within ten days of his seizure, and remained some days in each place.

*Dr. Cronyn* spoke of the case first mentioned by Dr. Rochester, he having had the charge of it subsequently. He had no doubt of the character of the disease. Had since had another case in the same family, for which he was called upon to prescribe, not however until it was too late to be of any assistance. This child died after a brief illness, covered with purple spots larger than petechiae.

Dr. C. also related a case of disease of the kidney in a lady, in whom a tumor made its appearance above the crest of the ilium. This lady had had pain in the back, for which she had been treated for five years previous. The urine became so scanty that but a small teacupful was passed in thirty-six hours. This, on testing, was found to be one-half albumen. Was still under treatment.

Under the head of "prevailing diseases," Drs. Rochester, Congar, Eastman, Lockwood, Wyckoff, Wetmore and Gay, spoke of the prevalence of diarrhœa, and some cases of cholera morbus. Dr. Eastman attributed these cases to the impure condition, this spring, of the Niagara water so generally used by our citizens. Drs. Wyckoff and Rochester dissented from this view, the former alluding to two cases in his practice in which no Niagara water had been used. Dr. Wetmore called attention to the character of the evacuations in the cases which had come under his notice, they being light colored and serous. Dr. Ring reported a case of typhoid fever, accompanied with severe diarrhœa, which he had seen.

*Dr. Eastman*, from the Committee on Vaccination, made some interesting statements on the subject, and urged upon physicians the importance of impressing their patients with the necessity for vaccination.

*Dr. Rochester* alluded to the subject of the condition of men liable to draft, and was inclined to believe our native born citizens, as a rule, more disqualified from various causes, than those of other nationalities.

*Dr. Trowbridge*, as Examining Surgeon Thirtieth District, N. Y., had had considerable opportunity for comparison, and could not agree with Dr. Rochester. Believed Americans, on the whole, at least equal in *physique* to foreigners. Would furnish some statistics soon from his official reports for the Association.

*Dr. Wetmore* proposed Dr. George U. Gleason for membership.

Adjourned. JOSEPH A. PETERS, Sec'y.

